# Is It Possible to Establish a Reliable Correlation between Maximum Standardized Uptake Value of 18-Fluorine Fluorodeoxyglucose Positron Emission Tomography/Computed Tomography and Histological Types of Non-Small Cell Lung Cancer? Analysis of the Italian VATS Group Database

**DOI:** 10.3390/diagnostics11101901

**Published:** 2021-10-14

**Authors:** Duilio Divisi, Marta Rinaldi, Stefano Necozione, Carlo Curcio, Federico Rea, Francesco Zaraca, Andrea De Vico, Gino Zaccagna, Gabriella Di Leonardo, Roberto Crisci, on behalf of the Italian VATS Group

**Affiliations:** 1 Thoracic Surgery Unit, Department of Life, Health & Environmental Sciences, University of L’Aquila, “Giuseppe Mazzini” Hospital, Piazza Italia 1, 64100 Teramo, Italy; martabrinaldi@gmail.com (M.R.); andrea.devico@aslteramo.it (A.D.V.); ginozcc@libero.it (G.Z.); gabriella.dileonardo@aslteramo.it (G.D.L.); roberto.crisci@univaq.it (R.C.); 2 Department of Internal Medicine and Public Health, University of L’Aquila, 67100 L’Aquila, Italy; stefano.necozione@univaq.it; 3 Division of Thoracic Surgery, Monaldi Hospital, 80131 Naples, Italy; carlo.curcio@ospedalideicolli.it; 4 Department of Cardiac, Thoracic, Vascular Sciences and Public Health, University of Padova, Padova City Hospital, 35100 Padova, Italy; federico.rea@unipd.it; 5 Department of Vascular and Thoracic Surgery, Central Hospital, 39100 Bolzano, Italy; francesco.zaraca@sabes.it

**Keywords:** NSCLC, biological framework, 18F-FDG-PET/CT, SUVmax, VATS group, data quality

## Abstract

Background. Although positron emission tomography/computed tomography, often integrated with 2-deoxy-2-[fluorine-18] fluorine-D-glucose (18F-FDG-PET/CT), is fundamental in the assessment of lung cancer, the relationship between metabolic avidity of different histotypes and maximum standardized uptake value (SUVmax) has not yet been thoroughly investigated. The aim of the study is to establish a reliable correlation between Suvmax and histology in non-small cell lung cancer (NSCLC), in order to facilitate patient management. Methods. We retrospectively assessed the data about lung cancer patients entered in the Italian Registry of VATS Group from January 2014 to October 2019, after establishing the eligibility criteria of the study. In total, 8139 patients undergoing VATS lobectomy were enrolled: 3260 females and 4879 males. The relationship between SUVmax and tumor size was also analyzed. Results. The mean values of SUVmax in the most frequent types of lung cancer were as follows: (a) 4.88 ± 3.82 for preinvasive adenocarcinoma; (b) 5.49 ± 4.10 for minimally invasive adenocarcinoma; (c) 5.87 ± 4.18 for invasive adenocarcinoma; and (d) 8.85 ± 6.70 for squamous cell carcinoma. Processing these data, we displayed a statistically difference (*p* < 0.000001) of FDG avidity between adenocarcinoma and squamous cell carcinoma. Moreover, by classifying patients into five groups based on tumor diameter and after evaluating the SUVmax value for each group, we noted a statistical correlation (*p* < 0.000001) between size and FDG uptake, also confirmed by the post hoc analysis. Conclusions. There is a correlation between SUVmax, histopathology outcomes and tumor size in NSCLC. Further clinical trials should be performed in order to confirm our data.

## 1. Introduction

Lung cancer remains in the leading causes of death for cancer worldwide and, despite the constant improvement of multidisciplinary treatment, carries a poor prognosis [1]. Video-assisted thoracoscopic surgery (VATS) provides the best chance of approach, especially for the early-stage bronchogenic carcinoma [2]. Non-small cell lung cancer (NSCLC) accounts for more than 80% of lung cancer and squamous lung cancer, compared to adenocarcinoma, which was associated with better prognosis [3]. The evaluation of computed tomography (CT) and positron emission tomography/computed tomography (PET/CT) findings are considered the cornerstones for diagnosis and staging in NSCLC, and according to 2017 Fleischner Society guidelines [4], these investigations are mandatory to assess the nature of lung lesions, both solid and part-solid ones. PET with 2-deoxy-2-[fluorine-18]fluoro-D-glucose integrated with computed tomography (18F-FDG-PET/CT) has proven to be significantly more accurate than computed tomography in the distinction between benign and malignant lesions and in the evaluation of metastatic spread [5,6], with a cutoff for malignancy ≥2.5 SUVmax value [7]. The correlation between tumor glucose uptake of FDG and other biological parameters predictive of tumor aggressiveness has recently been focused [8,9]. The maximum standardized uptake value (SUVmax), as a semiquantitative measurement of FDG uptake, is used to measure the uptake of FDG in the malignant tissue and provide intrinsic molecular-biological information of tumor. The intensity of FDG uptake of the primary tumor is highly correlated with disease recurrence and survival in NSCLC patients [10]. The possible implication of SUVmax to distinguish between different histological types of lung cancer (squamous cell carcinoma or adenocarcinoma) has not yet been properly studied, though pulmonary squamous cell carcinoma displays a higher glucose transporter type 1 (GLUT-1) expression and F-FDG uptake than adenocarcinoma [11]. Moreover, whether histological subtypes of adenocarcinoma also differ in F-FDG uptake is still not clear, and there is no evidence that clarifies if tumor size had a role in varying the degree of SUVmax absorption between the different types of lung cancer. In our study, we sought to evaluate the clinical effectiveness of 18F-FDG-PET/CT in the management of NSCLC patients, assessing the association between FDG-PET/CT avidity and the histological subtype of these lesions with postoperative outcomes, and its value in predicting tumor’s aggressiveness and histological type.

## 2. Material and Methods

### 2.1. Study Design

After approval by the Italian Registry of VATS Group, we retrospectively evaluated consecutive lung cancer patients whose data had been entered in the National Database between January 2014 and October 2019 and had received surgery. The data collected were from 57 Italian thoracic surgery centers affiliated to the VATS Group. We enrolled in the study only patients who had a preoperative 18F-FDG-PET/CT and who did not undergo neoadjuvant chemotherapy, in order to avoid any interference with the radiopharmaceutical uptake which is proportional to the glucose consumption by cells. This multicentric data collection was performed in order to evaluate the clinical effectiveness of 18F-FDG-PET/CT in the management of NSCLC patients. The eligibility criteria included: availability of complete imaging work-up, including whole body CT and PET/CT, assessment of the SUVmax, as index of the uptake of FDG by the lesion, surgical treatment and finally, availability of postoperative histological outcomes. In total, 8411 patients were recruited, although 272 patients did not meet inclusion criteria, and they did not undergo PET/CT scan. In total, 3260 females (40.1%) and 4879 (59.9%) males were enrolled (average age at surgical procedure, in 8139 patients, was 67.9 ± 9 years, range: 18–90 years). CT confirmed the nature of the lesions according to the evaluation of their morphology, density attenuation and pathological patterns: 6469 showed solid nature (79.5%), whereas 1670 (20.5%) displayed subsolid characteristic that includes 218 ground-glass opacities (GGOs) and 1452 part-solid nodules (PSNs). Preoperative PET/CT scan was performed, and SUVmax was measured to evaluate the FGD uptake. Demographic, clinical data and type of VATS lobectomy were summarized in Table 1. The ECOG (Eastern Cooperative Oncology Group) scale of performance status was Grade 0 in 4299 patients and Grade 1 in 3840 patients. On histological examination of the tissues surgically resected, 95.6% had a primary lung cancer (7783 patients), and 4.4% had secondary lung cancer (356 patients), as summarized in Table 2. According to the 2015 WHO Classification of Lung Cancer [12], the primary adenocarcinoma was the predominant histological type (71.1%), followed by squamous cell carcinoma (16.1%) and by neuroendocrine tumors and other carcinomas (10.2% and 2.6%, respectively).

### 2.2. PET/CT Image Acquisition

Since this is a multicenter study and each center is equipped with a different system, it is impossible to establish a uniform protocol. However, the PET images were acquired between 45 and 60 min after intravenous infusion of the ^18^F-FDG (between 5 and 7.5 MBq/kg), and the blood glucose level was constantly within the tolerance limits with maximum values of 180 mg/dL. The reconstruction of the images and their interpretation was carried out according to the criteria published by the European Association of Nuclear Medicine [13].

### 2.3. Endpoints of the Study

The primary endpoint of our study was to establish the association between SUVmax of PET/CT and histotype of lung cancer, in order to evaluate the efficacy of PET/CT to predict the histological feature of the lesions and to assess the role of SUVmax in discerning between frankly invasive lesions, commonly associated with a higher risk of metastatic spread, and minimally invasive or preinvasive lesions, correlated with a better patients’ survival. The secondary endpoint was to assess the association between SUVmax of PET/CT and tumor size, in order to establish how the size of the lesion could influence the uptake of FDG in different histological lesions, in case any significant differences were found between the previous cohorts. Thus, the aim of the study is focused on the possibility to establish the clinical effectiveness of PET/CT in predicting tumor’s aggressiveness and histotype, given the capability of SUVmax to analyze intrinsic factors of lung cancer.

### 2.4. Data Analysis

A dataset created with Numbers for macOS Catalina 10.15.4 (Apple Inc. Cupertino, CA, USA) was used to categorize patients’ characteristics collected from the VATS Group database. Statistical analysis was performed using the SAS—Statistical Analysis System for Windows (Microsoft, Redmond, Washington DC, USA). The descriptive analysis was expressed in terms of frequency, mean, median and standard deviation. Statistical differences or comparisons of three different parameters (SUVmax, histology and tumor size) were performed with nonparametric test, since the measurement of SUVmax is semiquantitative, and the distribution was not normal. Kruskal–Wallis test was used to estimate association between SUVmax and histological types, firstly considering all the histotypes collected, and subsequently only focusing on the relationship between SUVmax and the most common histological types of lung cancer: squamous cell carcinoma and adenocarcinoma with its subtypes (preinvasive, minimally invasive and invasive carcinoma). When a significant difference was found between SUVmax values and histological types of lung cancer, one-way analysis of variance (ANOVA test) was used to estimate the association between SUVmax and tumor size (T expressed in cm). The results were subsequently confirmed with post hoc analysis (Conover’s test). A *p* value less than 0.05 was considered to be significant in statistical analyses.

## 3. Results

**Figure 1:**
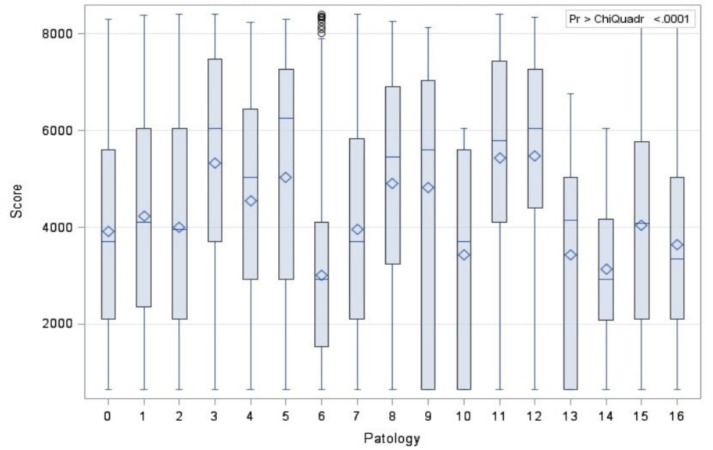
Correlation between SUVmax and histological types.

**Figure 2:**
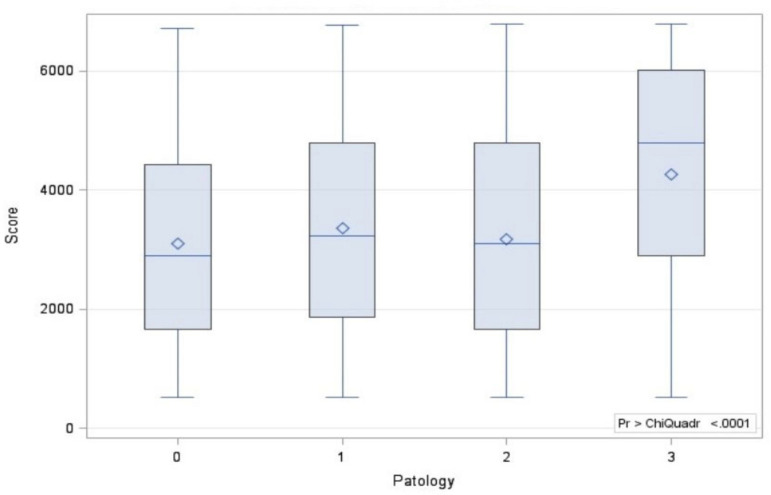
Correlation between SUVmax and the most common histological types of lung cancer.

**Figure 3:**
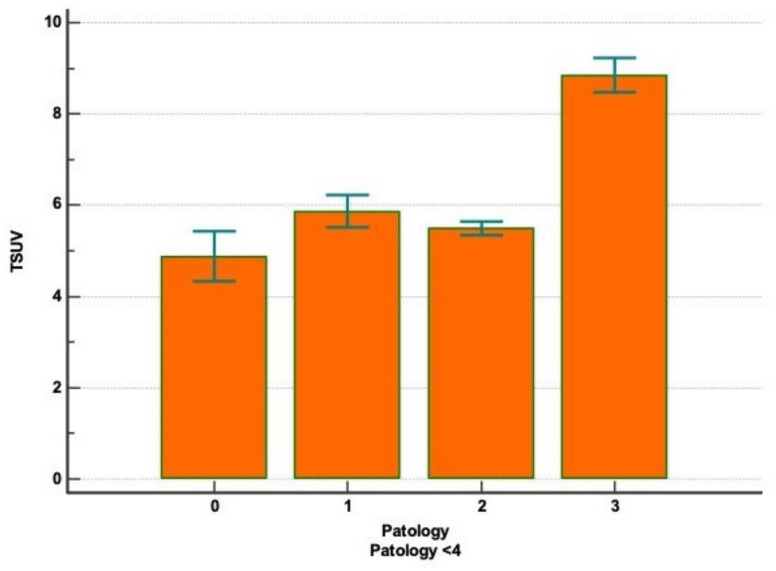
Correlation between SUVmax and pathology.

Histological subtypes of adenocarcinoma were classified in different cohorts, since they are not only relevant for their morphological significance but also for their biological and clinical implications. In fact, the preinvasive adenocarcinoma (187 patients) showed the most favorable prognosis, and the minimally invasive adenocarcinoma (767 patients) and invasive adenocarcinoma (4580 patients) displayed intermediate and strong aggressiveness, respectively. The descriptive analysis was summarized in Table 3. The subclassification of squamous cell carcinoma, further subdivided into keratinizing, non-keratinizing, and basaloid subtypes showed no apparent prognostic utility, and it was not taken into account in our study. The mean value of SUVmax was 6.0 (range, 0–78). A different SUVmax mean value was recorded for each adenocarcinoma subtype and for squamous cell carcinoma. Values expressed as mean ± standard deviation were as follows: (a) preinvasive adenocarcinoma, SUVmax 4.88 ± 3.82; (b) minimally invasive adenocarcinoma, SUVmax 5.49 ± 4.10; (c) invasive adenocarcinoma, SUVmax 5.87 ± 4.18; and (d) squamous cell carcinoma, SUVmax 8.85 ± 6.70. Evaluating CT and PET/CT findings, we classified patients between Path 0 and Path 5 into nine stages according to the Lung-Cancer TNM 8th Edition [14] (Table 4). Bivariate analysis between histology and SUVmax was calculated with Kruskal–Wallis test (Figure 1 and Figure 2), and the outcomes were confirmed by a post hoc analysis, represented in the Figure 3 and in Table 5. A statistical difference of FDG avidity was found between adenocarcinoma and squamous cell carcinoma (*p* < 0.000001). Regarding to adenocarcinoma subtypes, we displayed a significant difference of SUVmax value only between minimally invasive adenocarcinoma and invasive adenocarcinoma (*p* < 0.05).

**Figure 4:**
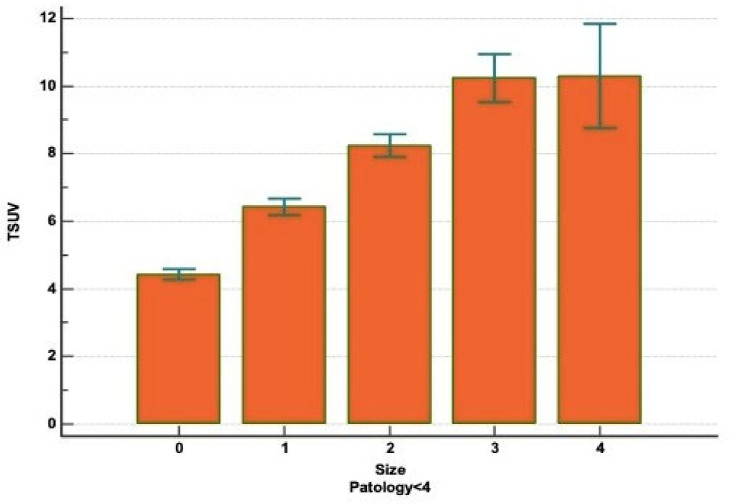
Correlation between SUVmax and tumor size.

The diameter of tumor size (T) was defined as the greatest transaxial dimension of the tumor in the lung window. Patients were classified into five groups depending to the size of the mass: (a) Group 0, patients with nodule diameter <2 cm; (b) Group 1, patients with nodule diameter between 2.1 and 3 cm; (c) Group 2, patient with nodule diameter between 3.1 and 5 cm; (d) Group 3, patients with nodule diameter between 5.1 and 7 cm; (e) Group 4, patients with nodule diameter >7 cm. The SUVmax values based on tumor size were as follows: (a) Group 0, SUVmax 4.39 ± 3.15; (b) Group 1, SUVmax 6.37 ± 3.45; (c) Group 2, SUVmax 8.19 ± 5.67; (d) Group 3, SUVmax 9.99 ± 6.61; and (e) Group 4, SUVmax 9.54 ± 5.55. The correlation between the T and the “SUVmax” parameters, in adenocarcinoma and squamous cell carcinoma patients, was carried out by one-way analysis of variance (Kruskal–Wallis test). In this regard, considering the SUVmax as a dependent variable and tumor size as an independent variable, a statistical correlation (*p* < 0.000001) between the two variables was identified (Figure 4). Moreover, a post hoc analysis conducted with Conover’s test (Table 6), confirmed our outcomes and highlighted the differences between the single groups. Statistical analysis according to the number of patients was not performed in Paths 4 and 5 and from Path 10 to Path 14 because are a simple statistically irrelevant. The metastatic cancers have not showed a statistically significant difference of FDG avidity compared to primary lung cancer (*p* = 3.5) and their different histologies (*p* = 4.3). Neuroendocrine (NE) tumors displayed an increased uptake of ^18^F-FDG from low-grade (typical carcinoid, SUVmax 3.83 ± 2.76) to higher-grade tumors (large cell NE carcinoma, SUVmax 8.64 ± 6.07) with statistical significance (*p* < 0.001).

## 4. Discussion

The relative great accuracy of PET/CT over conventional modalities, as computed tomography, for early detection of NSCLC and oncological staging has reported with increasing frequency. However, whether this method should be applied in daily practice remains under debate because of its extremely high cost and the effectiveness to add further information on the nature of isolated lung lesions [15,16]. Undoubtedly, PET/CT allows clinicians to focus on specific characteristics of lung lesions, which CT alone cannot provide [17]. The fundamental problem of thoracic surgeon concerns the evaluation of lesions that cannot be defined histologically preoperatively due to topography (nodules deeply indented in the parenchyma), morphology (aspect evoking or not of tumor) and dimensions (nodules around 1–2 cm in diameter). The lack of an alteration in glucose metabolism and of a hilar and mediastinal lymphadenopathy or enlargement does not exempt us from a surgical exploration of lung, with intraoperative histological examination. Unfortunately, the proposal of this diagnostic-therapeutic plan to a patient with normal or limited cardiorespiratory function for lung resection, very often clashes with the lack of consent, which underlies a clinical-radiological progression of the tumor. Therefore, considering that lobectomy associated with systematic lymph node dissection is the treatment of choice in NSCLC, the patient’s potential doubts must be reduced through a metabolic evaluation highly evocative for a specific type of lung cancer. Since NSCLC is a heterogenous group of carcinomas with different biological behavior and prognoses, the possibility of identifying early the tumor type has an important and fundamental impact on treatment and survival of patients. It is as obvious that structural imaging alone may not yield all the information necessary to fully characterize cancer patients, especially regarding the intrinsic factors of the lesions. By contrast, the FDG-PET, being a functional imaging method and indicating the specific distribution of ^18^F-FDG uptake by tissues, could determine the relationship between FDG uptake, histological type of cancer, biologic aggressiveness and prognosis in NSCLC. Up to now, it has been described that FDG avidity is not tumor-specific [18,19,20]. Our study, where a 2.5 cutoff of the SUVmax has been used to establish malignancy and has showed a relationship between FDG uptake and histopathology of the tumor; in fact, the SUVmax was significantly higher in squamous cell carcinomas compared to adenocarcinomas. This result is in agreement with our previous experience [11], in which a significant difference (*p* = 0.013), considering only the squamous carcinoma and invasive adenocarcinoma, was noted. The explanation can be sought in a greater expression of glucose transporter type 1 (GLUT-1) in squamous cell carcinomas than adenocarcinomas, as widely demonstrated in the literature [21,22,23,24]. Koh et al. [25] studied, in 269 patients treated with surgical resection for NSCLC, the following criteria: (a) the metabolic tumor volume (MTV) and total lesion glycolysis (TLG) values, by preoperative 18F-FDG-PET/CT; and (b) the expression of GLUT-1. Authors revealed that these parameters were higher in squamous cell carcinomas than adenocarcinomas. Particularly in the adenocarcinomas, the values of MTV, TLG and SUVmax were higher in GLUT-1 positive than GLUT-1 negative patients with reduction of 5-year overall survival. Moreover, our study revealed a significant correlation between minimally invasive adenocarcinoma/invasive adenocarcinoma and SUVmax value. By contrast, among preinvasive and invasive adenocarcinoma, no statistical correlation in SUVmax was found. This observation partly supports the finding of Nakamura et al. [26], who displayed a significant difference (*p* = 0.0001) among all the adenocarcinoma subtypes and FDG uptake. The outcomes probably reflected the different grade of consolidation of these lesions. In fact, invasive adenocarcinoma displays a larger solid component compared to minimally invasive adenocarcinoma which shows the radiological features of a PSN with a small solid component. This last aspect is the expression of its lower invasiveness and aggressiveness [27]. Chiu et al. [28], studying 152 patients with 153 primary adenocarcinomas of the lung, also morphologically classified this NSCLC as terminal respiratory unit (TRU) type and non-TRU type. The TRU-type adenocarcinoma is characterized by TTF-1 (thyroid transcription factor-1) expression and EGFR (epidermal growth receptor factor) mutations. Authors highlighted that the GLUT-1 expression was higher in adenocarcinomas with solid component and non-TRU type than those without solid grow pattern (*p* = 0.002) and TRU type (*p* = 0.001) tumors. The above considerations are in agreement with our findings: in fact, our study showed a lower degree of SUVmax for preinvasive adenocarcinoma, predominantly occurred as GGOs, in comparison to minimally invasive or invasive adenocarcinoma. In addition, a high SUVmax may be correlated with programmed death ligand 1 (PD-L1) expression in adenocarcinoma patients, as demonstrated by Hu et al. [29], which is directly in turn linked to GLUT-1, such as showed by Cui et al. [30].

The secondary endpoint of the study was the evaluation of relationship between tumor size and the different degree of FDG uptake in the various histological types of NSCLC. As already reported, the higher FDG uptake in squamous cell carcinoma seems to be correlated with more rapid lung cancer proliferation and more rapid tumor doubling time (92 days for SCC and 168 for ADC) and with the consistent overexpression of GLUT-1. This last feature is linked to the bigger dimensions of squamous cell carcinoma, compared to adenocarcinoma, and to the hypoxic status, in which lung cells remain as the tumor increases in size and determines a greater expression of GLUT-1 transporter [31]. We found a statistically significant correlation between tumor size and SUVmax (*p* < 0.000001) in all four histologies of lung carcinoma. Moreover, evaluating the results of the post-hoc analysis, conducted with the Conover’s test, we displayed that considering only the Group 0, 1 and 2, statistical correlation in SUVmax was noted (*p* < 0.05), but, on the other hand, no significant difference was experienced between the Group 3 and 4. These data suggested that the SUVmax had a low clinical effectiveness in discerning between tumor with a diameter among 5.1–7 cm or greater than 7 cm. Thus, both histologic subtypes and tumor size have influence upon FDG uptake of primary tumor in NSCLC, especially in tumors with a transaxial diameter less than 5 cm. Finally, our findings concerning NE tumors confirmed that neoplasms with high proliferative and low differentiation cell turnover show greater uptake of FDG. Song et al. [32], studying 32 patients with NE lung tumor, highlighted that the higher GLUT-1 expression was associated with greater FDG avidity, proving the correlation between this trans-membrane protein and glucose uptake also in the neuroendocrine histotypes. However, the current trend is to use the different ^18^F-FDG and ^68^Ga uptake to define the degree of NE tumor aggressiveness [33]. There may be three possible study biases. The first is that we only retrospectively considered lung cancer patients, excluding any other pathology that could serve as a yardstick of comparison. The second is that patients were super-selected based on the indications for the VATS resection. The excellent performance status probably does not allow one to reach definite conclusions, as the influence on glucose uptake by an associated lung disease is still unclear. The third is the use of different PET/CT scanners with various settings, although the reference values and image acquisition times have been standardized.

## 5. Conclusions

The correlation between SUVmax, histopathology outcomes and tumor size in NSCLC, especially between squamous cell carcinomas and adenocarcinomas, partly interprets the capability of SUVmax to predict, in the preoperative assessment of lung mass, the histology of the lesion and its invasiveness. Although our study suggests that FDG-PET intensity may be a way to assess the biological aggressiveness and prognosis of lung cancer, further systematic investigations and research are needed before this can be broadly applied clinically. Indeed, the literature has so far given conflicting results. Moreover, our study highlighted how the role of FDG uptake in prognosis stratification can be another exciting development area that should be precisely determined in the near future by the prospective randomized clinical trials, with the greatest number of patients possible.

## Figures and Tables

**Table 1:** Demographics, radiological and pathological characteristics.

Clinical Data	N	%	Mean	Interval
Gender				
Male	4879	59.9%		
Female	3260	40.1%		
Age			67.9	18–90
Nodule Size (CT)				
Group 0 (T < 2 cm)	3869	47.6%		
Group 1 (2.1 < T > 3 cm)	2198	27%		
Group 2 (3.1 < T > 5 cm)	1669	20.5%		
Group 3 (5.1 < T > 7 cm)	345	4.2%		
Group 4 (T > 7 cm)	58	0.7%		
Nodule Density (CT)				
Solid	6469	79.5%		
Part-solid	1452	17.8%		
Pure GGO	218	2.7%		
SUVmax (PET)			6.19	0–78
Type of lesion				
Malignant (primary)	7783	95.6%		
Malignant (secondary)	356	4.4%		
Type of resection				
Upper Lobectomy	4407	54.1%		
Middle Lobectomy	590	7.3%		
Lower Lobectomy	2742	33.7%		
Upper Bilobectomy	69	0.9%		
Lower Bilobectomy	49	0.6%		
Basal Segmentectomy	39	0.5%		
Apical segmentectomy of upper lobe	99	1.2%		
Apical segmentectomy of lower lobe	83	1.0%		
Lingulectomy	61	0.7%		

**Table 2:** Histological types according to the “2015 WHO Classification of Lung Tumors” [12].

	Types	N	%
	Adenocarcinoma *		
Path 0	Preinvasive lesion	187	2.3%
Path 1	Minimally invasive	767	9.4%
Path 2	Invasive Adenocarcinoma	4580	56.3%
Path 3	Squamous Cell Carcinoma *	1254	15.4%
Path 4	Adenosquamous Cell Carcinoma	90	1.1%
Path 5	Pleomorphic Carcinoma	33	0.4%
	Neuroendocrine Tumors *		
Path 6	Typical Carcinoid	448	5.5%
Path 7	Atypical Carcinoid	107	1.3%
Path 8	Small Cell Lung Cancer	60	0.7%
Path 9	Large Cell Neuroendocrine Carcinoma	180	2.2%
	Other Carcinomas *		
Path 10	NSCLC NAS	23	0.3%
Path 11	Mucoepidermoid Carcinoma	7	0.1%
Path 12	Carcinosarcoma	26	0.3%
Path 13	Adenoidocystic Carcinoma	13	0.2%
Path 14	Lymphoma *	8	0.1%
Path 15	Metastatic Cancer *	356	4.4%

* The bold format identifies the histological types found.

**Table 3:** Descriptive analysis with SUVmax as dependent variable, based on histologic diagnosis. The analysis was expressed in terms of frequency, mean, median and standard deviation. All the histological types of lung cancer, collected in our study, were reported. Path 0, Preinvasive Adenocarcinoma; Path 1, Minimally invasive Adenocarcinoma; Path 2, Invasive Adenocarcinoma; Path 3, Squamous Cell Carcinoma; Path 4, Adenosquamous Cell Carcinoma; Path 5, Pleomorphic Carcinoma; Path 6, Typical Carcinoid; Path 7, Atypical Carcinoid; Path 8, Small Cell Lung Cancer; Path 9, Large Cell Neuroendocrine Carcinoma; Path 10, NSCLC NAS; Path 11, Mucoepidermoid Carcinoma; Path 12, Carcinosarcoma; Path 13, Adenoidocystic Carcinoma; Path 14, Lymphoma; Path 15, Metastasis.

	N	Mean	St. Dev	Median	Min	Max
Path 0	187	4.88	3.82	4.00	0.00	24.00
Path 1	767	5.49	4.10	4.10	0.00	31.00
Path 2	4580	5.87	4.18	4.70	0.00	45.00
Path 3	1254	8.85	6.70	8.00	0.00	51.00
Path 4	90	6.55	5.39	6.00	0.00	21.00
Path 5	33	8.15	6.47	8.50	0.00	24.00
Path 6	448	3.83	2.76	3.00	0.00	78.00
Path 7	107	6.41	3.48	4.00	0.00	77.00
Path 8	60	7.13	5.05	6.65	0.00	21.46
Path 9	180	8.64	6.07	7.27	0.00	39.00
Path 10	23	7.11	5.51	7.00	0.00	18.39
Path 11	7	3.86	3.13	4.00	0.00	8.00
Path 12	26	9.36	7.30	8.00	0.00	28.00
Path 13	13	4.00	3.39	4.80	0.00	10.00
Path 14	8	3.41	2.56	3.00	0.00	8.00
Path 15	356	5.57	3.57	4.60	0.00	31.00

**Table 4:** Clinical staging (6911 Path 0–Path 5 patients) according to the “International Association for the study of Lung Cancer—Stage Grouping for the 8th Edition of the TNM Classification for Lung Cancer” [14].

STAGE	N	%
IA1	2148	31.1%
IA2	1868	27%
IA3	715	10.3%
IB	1061	15.3%
IIA	190	2.8%
IIB	580	8.4%
IIIA	301	4.4%
IIIB	45	0.7%
IIIC	3	0%

**Table 5:** Post hoc analysis (Conover) of adenocarcinomas and epidermoid carcinomas.

Pathology	N	Average Rank	Different (*p* < 0.05) from Factor Nr.
Path 0	187	3094.74	3
Path 1	767	3357.34	2–3
Path 2	4580	3177.57	1–3
Path 3	1254	4254.24	0–1–2

Path 0, Preinvasive Adenocarcinoma; Path 1, Minimally Invasive Adenocarcinoma; Path 2, Invasive Adenocarcinoma; and Path 3, Squamous Cell Carcinoma.

**Table 6:** Post hoc analysis (Conover) of adenocarcinomas and epidermoid carcinomas based on diameter of lesion.

Figure	N	Average Rank	Different (*p* < 0.05) from Factor Nr.
0 (<2 cm)	3052	2788.82	1–2–3–4
1 (between 2.1 and 3 cm)	1876	3651.76	0–2–3–4
2 (between 3.1 and 5 cm)	1464	4083.42	0–1–3–4
3 (between 5.1 and 7 cm)	323	4729.68	0–1–2
4 (>7 cm)	73	4694.71	0–1–2

## Data Availability

Data can be found in the Italian VATS Group database (https://www.vatsgroup.it/).

## Italian VATS Group Collaborator List

Duilio Divisi, Prof (University of L’Aquila, “G. Mazzini” Hospital, Teramo); Marta Rinaldi, MD (University of L’Aquila, “G. Mazzini” Hospital, Teramo); Carlo Curcio, MD (Monaldi Hospital, Napoli); Roberto Crisci, Prof (University of L’Aquila, “G. Mazzini” Hospital, Teramo); Marco Alloisio, MD (IRCCS Humanitas Milano); Dario Amore, MD (Monaldi Hospital, Napoli); Luca Ampollini, MD (University of Parma); Nicoletta Ardò, MD (Riuniti Hospitals, Foggia); Desideria Argnani, MD (AUSL Romagna Teaching Hospital, Forlì); Alessandro Baisi, MD (S. Paolo Hospital, Milano); Alessandro Bandiera, MD (San Raffaele Hospital, Milano); Cristiano Benato, MD (Borgo Trento Hospital, Verona); Mauro Roberto Benvenuti, MD (Spedali Civili Hospital, Brescia); Alessandro Bertani, MD (IRCCS ISMETT, Palermo); Luca Bertolaccini, MD, PhD (IEO Hospital, Milano); Luigi Bortolotti, MD (Humanitas Gavazzeni Hospital, Bergamo); Edoardo Bottoni, MD (IRCCS Humanitas, Milano); Cristiano Breda, MD (Dell’Angelo Hospital, Mestre); Pierpaolo Camplese, MD (S. Maria Annunziata Hospital, Chieti); Paolo Carbognani, MD (University of Parma); Giuseppe Cardillo, MD (Forlanini Hospital, Roma); Francesco Carleo, MD (Forlanini Hospital, Roma); Giorgio Cavallesco, MD (University of Ferrara); Roberto Cherchi, MD (Businco Hospital, Cagliari); Andrea Dell’Amore, Prof (University of Padova); Vittorio Della Beffa, MD (San Giovanni Bosco Hospital, Torino); Angela De Palma, MD (Giovanni XXIII Hospital, Bari); Giampiero Dolci, MD (S. Orsola Hospital, Bologna); Andrea Droghetti, MD (ASST Mantova-Cremona, Mantova); Paolo Albino Ferrari, MD (Businco Hospital, Cagliari); Diego Fontana, MD (San Giovanni Bosco Hospital, Torino); Roberto Gasparri, MD (IEO Hospital, Milano); Diego Gavezzoli, MD (Spedali Civili Hospital, Brescia); Marco Ghisalberti, MD (University of Siena); Michele Giovanardi, MD (ASST Mantova-Cremona, Mantova); Alessandro Gonfiotti, MD (Careggi Hospital, Firenze); Francesco Guerrera, MD (Molinette Hospital, Torino); Andrea Imperatori (University of Varese); Paolo Lausi, MD (Molinette Hospital, Torino); Fabio Lo Giudice, MD (Dell’Angelo Hospital, Mestre); Francesco Londero, MD (S. Maria Della Misericordia Hospital, Udine); Camillo Lopez, MD (Vito Fazzi Hospital, Lecce); Luca Luzzi, MD (University of Siena); Maurizio Mancuso, MD (S.S. Antonio e Biagio e Cesare Arrigo Hospital, Alessandria); Pio Maniscalco, MD (University of Ferrara); Stefano Margaritora, Prof (Gemelli Hospital, Roma); Federico Mazza, MD (Santa Croce Hospital, Cuneo); Elisa Meacci, MD (Gemelli Hospital, Roma); Giulio Melloni, MD (Santa Croce Hospital, Cuneo); Angelo Morelli, MD (S. Maria Della Misericordia Hospital, Udine); Felice Mucilli, MD (S. Maria Annunziata Hospital, Chieti); Pamela Natali, MD (University of Modena); Giampiero Negri, MD (San Raffaele Hospital, Milano); Samuele Nicotra, MD (University of Padova); Mario Nosotti, Prof (University of Milano, Ca’Granda Hospital); Gian Luca Pariscenti, MD (San Martino Hospital, Genova); Reinhold Perkmann, MD (Bolzano Hospital, Bolzano); Fausto Pernazza, MD (S.S. Antonio e Biagio e Cesare Arrigo Hospital, Alessandria); Emanuele Pirondini, MD (San Gerardo Hospital, Monza); Francesco Puma, MD (University of Perugia); Federico Raveglia, MD (San Paolo Hospital, Milano); Mayed Refai, MD (Riuniti Hospital, Ancona); Ottavio Rena, MD (Maggiore Della Carità Hospital, Novara); Alessandro Rinaldo, MD (Niguarda Hospital, Milano); Carlo Risso, MD (San Martino Hospital, Genova); Giovanna Rizzardi, MD (Humanitas Gavazzeni Hospital, Bergamo); Nicola Rotolo, MD (University of Varese); Marco Scarci, MD (San Gerardo Hospital, Monza); Piergiorgio Solli, MD (S. Orsola Hospital, Bologna); Francesco Sollitto, Prof (Riuniti Hospitals, Foggia); Lorenzo Spaggiari, MD (IEO Hospital, Milano); Alessandro Stefani, MD (University of Modena); Franco Stella, MD (AUSL Romagna Teaching Hospital, Forlì); Corrado Surrente, MD (Vito Fazzi Hospital, Lecce); Giorgia Tancredi, MD (IRCCS ISMETT, Palermo); Alberto Terzi, MD (Negrar Hospital, Verona); Massimo Torre, MD (Niguarda Hospital, Milano); Damiano Vinci, MD (University of Perugia); Andrea Viti, MD (Negrar Hospital, Verona); Luca Voltolini, MD (Careggi Hospital, Firenze); and Francesco Zaraca, MD (Bolzano Hospital, Bolzano).
